# Oxidative Stress and Mitochondrial Dysfunction in Down's Syndrome: Relevance to Aging and Dementia

**DOI:** 10.1155/2012/383170

**Published:** 2012-04-29

**Authors:** Pinar E. Coskun, Jorge Busciglio

**Affiliations:** ^1^Department of Neurobiology and Behavior, Institute for Memory Impairments and Neurological Disorders (iMIND), University of California, Irvine, CA 92697, USA; ^2^Center for the Neurobiology of Learning and Memory (CNLM), University of California, Irvine, CA 92697, USA

## Abstract

Genome-wide gene deregulation and oxidative stress appear to be critical factors determining the high variability of phenotypes in Down's syndrome (DS). Even though individuals with trisomy 21 exhibit a higher survival rate compared to other aneuploidies, most of them die *in utero* or early during postnatal life. While the survivors are currently predicted to live past 60 years, they suffer higher incidence of age-related conditions including Alzheimer's disease (AD). This paper is centered on the mechanisms by which mitochondrial factors and oxidative stress may orchestrate an adaptive response directed to maintain basic cellular functions and survival in DS. In this context, the timing of therapeutic interventions should be carefully considered for the successful treatment of chronic disorders in the DS population.

## 1. Introduction

Down's syndrome (DS) or trisomy 21 is a prevalent genetic cause of intellectual disability due to full or partial triplication of chromosome 21 (HSA21). The presentation varies greatly between individuals. The molecular bases of this variation is “the gene dosage effect” caused by the extra chromosome 21, which leads to a global imbalance on gene expression [1]. However, the molecular mechanisms by which such gene dosage imbalance causes DS-specific abnormalities remain unclear.

Albeit trisomy 21 is the most common aneuploidy that infants can survive, the rate of miscarriage of fetuses with DS during the first trimester is almost 50% [2]. The survival rate for the first 18 years of life of DS individuals is 50.3% of the total DS population, and the greatest percent of deaths is observed during the first 5 years of life (35.9%). The death rate drops to 13.1% between 19 and 40 years, and DS individuals of 40+ years have a greater chance to live beyond 60 years of age in developed countries, especially those without congenital heart disease [3].

A remarkable feature of the syndrome is the presence of Alzheimer's disease (AD) neuropathology in the brain of nearly all DS individuals, the majority of which develop dementia with age [4]. Besides dementia, other aging features appear prematurely such as cataracts, diabetes, hair graying, leukemia, and hearing and visual impairment. Together, they define DS as a “segmental progeroid syndrome” [5–7]. Mitochondria represent both a principal source as well as a target of free radicals, which in turn cause structural damage and activate signaling pathways associated with ageing and age-related diseases [8–10]. Both oxidative stress and mitochondrial dysfunction are prominent features of DS [11–14]. The relation between oxidative stress, genome imbalances, specific HSA21 genes, and the DS phenotype has been discussed elsewhere [11, 14–17]. In this paper, we will primarily focus on mitochondrial deregulation, oxidative stress, and the emergence of an adaptive response, which may influence the timing and extent of clinical manifestations in DS.

## 2. Mitochondria and Oxidative Stress

Mitochondria have three major functions: generation of ATP, production of reactive oxygen species (ROS) and initiation of apoptosis. NADH and FADH_2_ formed in glycolysis, fatty acid oxidation, and the citric acid cycle are used to reduce oxygen to water by a series of electron carriers located in the inner mitochondrial membrane. The flow of electrons leads to the pumping of protons out of the mitochondrial matrix and the formation of a proton gradient across the inner membrane, which provides the driving force used by ATP synthase to produce ATP. This process is known as oxidative phosphorylation (OXPHOS) [18, 19]. Most of the cellular ROS are produced by electrons escaping from the electron transport chain (ETC) which are captured by O_2_. Some studies suggest that as much as 2–5% of the total O_2_ intake ends up forming superoxide radicals. These are scavenged by antioxidant enzymes such as mitochondrial superoxide dismutase (SOD2) and glutathione peroxidase (Gpx) [20]. Mitochondrial DNA (mtDNA) encodes 37 genes: 13 mRNAs for subunits of ETC complexes, 22 tRNAs, and 2 rRNAs operating protein translation in the mitochondrial matrix [18]. Since mtDNA is in close proximity to the ETC, it can suffer mutations under excessive ROS production, leading to impaired gene expression and further reductions in ETC efficiency. Mitochondria eventually become dysfunctional beyond repair and lose their electrochemical membrane potential (MMP). The loss of MMP activates the permeability transition pore, releasing mitochondrial material to the cytoplasm. Ultimately, this triggers the execution phase of the apoptotic process [18], which has been implicated in multiple conditions including mitochondrial diseases, DS, and age-related neurodegeneration [21, 22].

Besides mtDNA, nuclear DNA (nDNA) encodes approximately 1600 mitochondrial genes [18, 21]. Because of the split location of mitochondrial genes, mitochondrial genetics does not follow Mendelian rules. While mitochondria and mtDNA are maternally inherited [21], nuclear encoded mitochondrial genes (NEMGs) are inherited from both parents. Since each cell has thousands of mitochondria and mtDNA copies; individual differences in the ratio of normal and mutant mtDNA lead to heteroplasmy. Variations in heteroplasmy and in the energy requirements of specific cells and tissues dictate the variability in the presentation of mitochondrial diseases, not unlike what is observed in DS. The proportion of mutated mtDNAs varies spatially (depending on the cell and tissue) and temporally (over the individual's life). Thus, a particular mtDNA mutation may cause variable phenotypes [18, 23]. For example, the mtDNA mutation tRNA^Leu^ A3243G has been associated with mitochondrial myopathy, encephalopathy, lactic acidosis, stroke-like episodes (MELAS), diabetes mellitus, Leigh's disease, and progressive external ophthalmoplegia (PEO) [24]. This variability in phenotypes may also be relevant to DS, where there is a high rate of mtDNA mutations and several mitochondrial genes are disproportionally expressed.

## 3. Mitochondria in DS and DSAD

**Figure 1:**
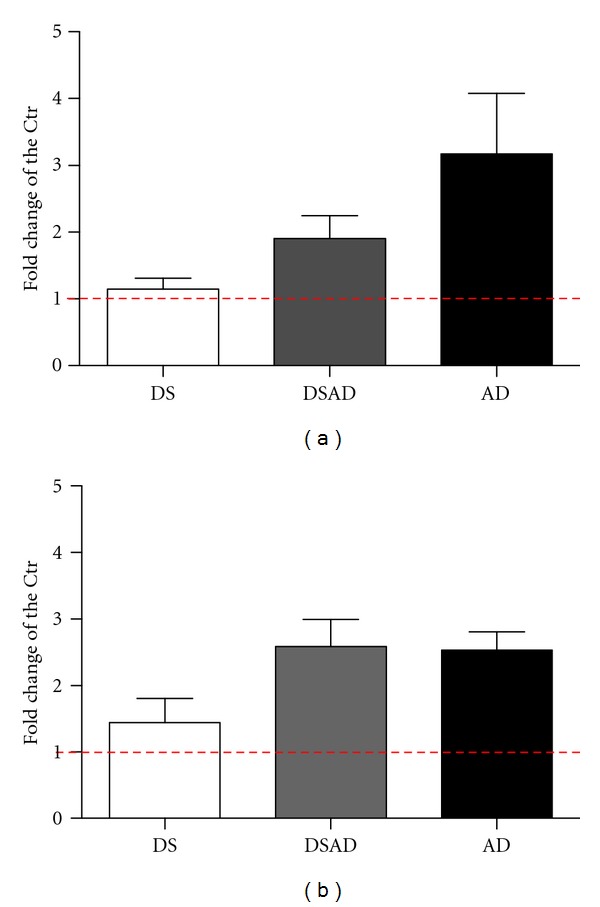
Accumulation of mitochondrial DNA mutations in DS, DSAD, and AD frontal cortex (a) and lymphoblastoid cell lines (LCL) (b). The graph was plotted as fold difference with respect to age-matched controls. DS brains age group: 0–40, DSAD age group: 45–68, and AD age group: 65–90. For each group 6 to 16 samples were analyzed. LCL lines for all groups (DS, DSAD, DAD, and control) were obtained from 40–60 years old donors, 6–8 samples per group. The red line shows the baseline mutation level for the control group.

In addition to a handful of mitochondrial genes in HSA21 whose deregulation may impair mitochondrial function, the evidence suggests that cytoplasmic inheritance of deleterious mtDNA mutations in maternal mitochondria can influence the frequency of DS in families or increase DS incidence in pregnancies from older age females [25, 26]. Mitochondrial activity is essential for spindle formation and chromosome segregation during meiosis and early embryogenesis [27]. Age appears to influence mitochondrial function in oocytes and follicular cells, and mtDNA mutations in oocytes have been found to be age related [23, 27]. Dysfunctional mitochondria have been implicated in the predisposition to chromosomal nondisjunction during the first and second meiotic divisions, in mitotic errors in embryos, and in the reduced quality and developmental potential of aged oocytes and embryos [23, 27, 28]. Thus, variable levels of mtDNA mutations in maternal mitochondria would be present in different DS individuals. Since mtDNA mutations accumulate with age, individuals starting their lives with higher mtDNA mutation rates would be more predisposed to age-related dementia. In fact, DS with Alzheimer's disease (DSAD) exhibit higher rates of mtDNA mutations in frontal cortex compared to DS and age-matched controls (Figure 1(a)).

**Figure 2:**
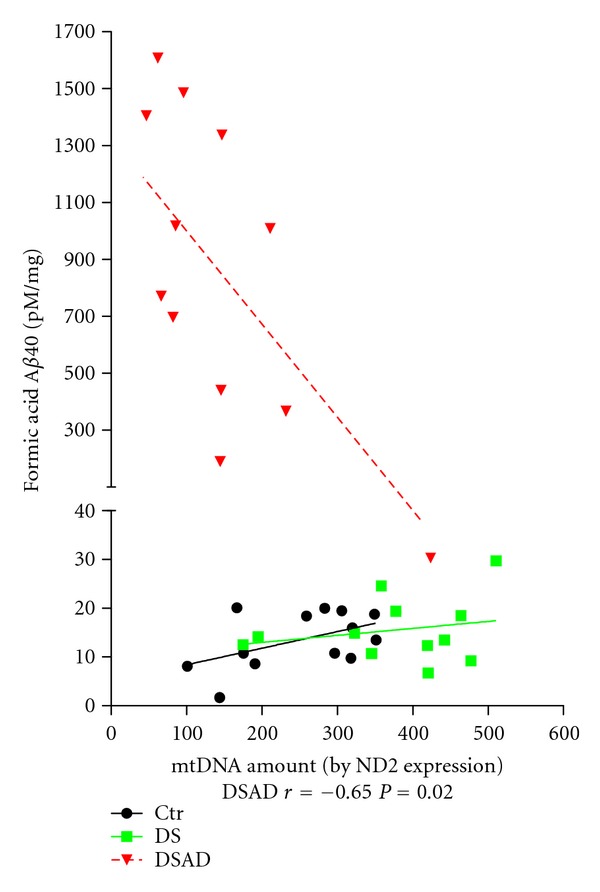
Levels of A*β* correlated with mitochondrial biogenesis represented as mtDNA amount. There was a significant inverse correlation between insoluble A*β* and mitochondrial DNA amount only in DSAD cases. Results reprinted from [29].

Similar differences were observed when mtDNA mutations were analyzed in lymphoblastoid cells (LCL) (Figure 1(b)) [29], indicating a systemic increase in mtDNA mutations in DSAD. Specific mtDNA nucleotides were mutated at higher rates in DSAD and sporadic AD than in controls, and mutations in replication and transcription regulatory sequences resulting in reduced mtDNA levels and light strand gene expression were found in brains of DSAD and AD individuals [29, 30]. Consistent with these studies, a previous report indicates defective repair of mtDNA damage in DS [31]. Interestingly, DS brains without AD exhibited a slight increase in mtDNA levels, suggesting a compensatory upregulation of mitochondrial biogenesis, which disappeared in DSAD subjects. This decrease in mitochondrial biogenesis with dementia correlates with increased Aß levels and deposition, suggesting A*β*-related toxic mechanisms affecting mitochondrial biogenesis (Figure 2) [29].

## 4. Oxidative Stress and Mitochondrial Alterations

Increased oxidative stress in DS and AD correlates with a decrease in several mitochondrial components including complex IV nuclear encoded subunit IV, mtDNA encoded subunit I [32], complex I nuclear encoded 24 and 75 kDa subunits [33], complex V nuclear encoded *β* subunit and complex III nuclear encoded core protein I [23], and mitochondrial ATPase6 and mitochondrial transcription factor A (Tfam) [34]. A recent study in DS fibroblasts found a specific deficiency in complex I, increased levels of several ETC components, and increased porin levels, further suggesting that mitochondrial biogenesis is upregulated in DS. The defect in complex I was associated with decreased cAMP-dependent phosphorylation of complex I 18 kDa subunit, reduced protein kinase A activity and low basal levels of cAMP. Mitochondrial superoxide production and oxidative stress were found to be 3 times higher in DS fibroblast, which were rescued by treatment with a cAMP analog [35]. The general changes in expression of mitochondrial enzymes correlate with a downregulation of the major mitochondrial heat shock protein, HSP60 [36, 37], which is critical to prevent protein aggregation during thermal and ROS stress. In addition, a number of mitochondrial proteins are elevated in DS including mitochondrial aconitase, NADP-linked isocitrate dehydrogenase [38], and the mitochondria-targeted ES1 protein homologue [39], all of which may be part of a compensatory antioxidant response to increased mitochondrial ROS production.

## 5. DS and Hormesis

Based on the considerations above, it is conceivable that oxidative stress and redox changes play a dual role in DS. At low levels, they promote cellular proliferation, while at higher levels, they produce oxidative damage and initiate apoptosis [40]. Adaptive response signaling, also known as hormesis, is triggered by sublethal stress, which stimulates cellular functional changes to protect against a subsequent exposure to more severe stress [41]. Consequently, compensatory mechanisms can prepare the cell to resist higher stress levels [42].

Since mitochondria are the main source of ROS production, their role is essential in age-related oxidative damage. While abundant research supports the idea that reduced oxidative stress is associated with increased life span [43–46] several experiments showed inconsistent or even contradictory results in human studies when interventions aimed to lower ROS level [47] were unable to produce health beneficial effects [48, 49]. In a recent example, which is relevant to DS, a 2-year randomized placebo-controlled daily oral antioxidant supplementation did not improve cognitive functioning nor it stabilized cognitive decline in DSAD [50]. These findings suggest that mitochondrial ROS production could indeed trigger cellular processes that promote health and longevity. Such signaling events, or adaptive response, are observed in the context of caloric restriction (CR), one of the best intervention strategies to increase life span from yeast to mammals. In fact, CR induces mitochondrial hormesis (mitohormesis) [51] by increasing mitochondrial respiration and elevating mitochondrial ROS production without changing ATP production [52].

A prominent sensor related to hormesis is the Keap1-Nrf2-ARE signaling complex. Under normal redox conditions, the transcription factor NFE2-related factor 2 (Nrf2) binds to Kelch-like ECH-associated protein 1 (Keap1) in the cytosol leading to its proteasomal degradation [53]. Keap1 is a cysteine-rich protein that senses redox changes in the cell. Under oxidative stress, conformational changes in Keap1 lead to its dissociation from the Nrf2-Keap1 complex and to the translocation of free Nrf2 into the nucleus, where it binds to antioxidant response element (ARE) regions in the genome, and activates the expression of stress response genes [54, 55]. So far, there is no complete information on Nrf2/Keap1 genes, protein levels or activities in DS. However, a recent study comparing gene expression profiles in DS and euploid astrocytes found that Nrf-2-associated oxidative stress response genes were differentially regulated in DS, supporting the presence of hormesis in DS [56]. Additional evidence of hormesis in DS comes from experiments showing increased activity of mitogen-activated protein kinases (MAPKs), including ERK1/2, SAPKs, and p38 in DS and AD brains [57]. MAPKs phosphorylate Nrf2 enabling its dissociation from the Nrf2/Keap1 complex [58]. *GPx* and *catalase* are also Nrf2 target genes carrying ARE sequences in their promoters [59]. Interestingly, higher intellectual function in DS correlated with increased expression of GPx, which could be part of the adaptive response in those individuals [60].

Nrf2 also interacts with PPAR*γ*, PGC1*α*, and PI3K/Akt, all of which participate in mitochondrial biogenesis [61]. Thus, these factors may underlie mtDNA increase [29] and mitochondrial biogenesis [35] in DS cells. Finally, a generalized downregulation of mitochondrial activity has been observed in different DS cell types including neurons, astrocytes, pancreatic *β* cells, endothelial cells, and fibroblasts, which is consistent with a cellular adaptation to reduce ROS production and prevent cellular injury [56]. However, additional stressors and/or challenges in the form of infections, seizures, age-related loss of function, and so forth can eventually exhaust the capacity of the functional adaptations to avert cellular damage. In this context, chronic respiratory infections and multiple signs of early senescence such as cataracts, skin atrophy, seizures, leukemia, and AD-type neuropathology may be the result of oxidative stress, mitochondrial dysfunction, and additional factors acting systemically or in specific organs and tissues. Thus, the severity of the DS phenotype may be the result of the initial level of mitochondrial mutations, the accumulation of oxidative damage, and the magnitude of the cellular adaptations triggered by these changes. Activation of an early adaptive response by initial sublethal levels of stress may translate in a longer survival. However, the combination of chronic stress and age-related changes would result in the premature and accelerated development of age-related conditions such as dementia and AD pathology.

One consequence of the considerations above is that not only the compounds but also the timing of treatment options should be carefully considered in DS patients. For example, long-term treatments designed to reduce oxidative stress may not add any incremental benefit on top of the changes driven by hormesis. Interventions would be more effective if introduced at the very onset of stress or disease. In fact, recent findings indicate that exercise-induced oxidative stress ameliorates insulin resistance and generates an adaptive response enhancing the endogenous antioxidant defense capacity [62]. However, supplementation with antioxidants may preclude the health-promoting effects of exercise in humans [62]. Thus, under normal conditions, antioxidants may not help and may even interfere with hormesis. According to this hypothesis, they would be most effective when an additional stressor is present.

**Figure 3:**
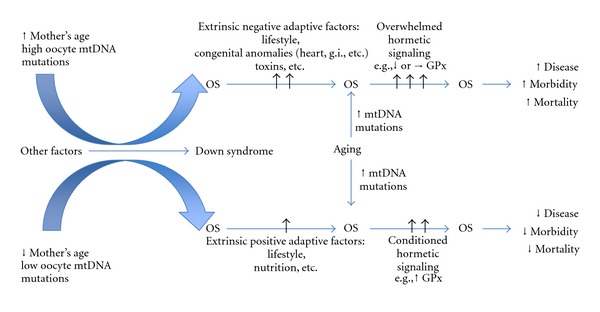
Modulation of DS phenotypes by oxidative stress and mitochondrial factors. Fetal oxidative stress (OS) levels could be determined by the mother's age and initial mtDNA mutation levels in oocytes. Besides the genetic/intrinsic factors that create the genomic instability in DS, environmental factors and lifestyle modulate the initial OS further. Since all these factors that play a role in the level of OS differ individually, the OS-related changes will also be observed in variation. Simply, while the low level of OS could initiate the positive adaptive response by activating proper defense signaling, high level of OS will start destructive signaling where the adaptive response could not be able to accommodate the clearance of the damage. More positive factors (e.g., lifestyle, advantageous genetic background—mitochondrial haplotype, APOE, BDNF genotype, etc.—and nutrition) will feed the adaptive response positively, while negative factors (e.g., congenital defects, sedentary lifestyle, genotypes, etc.) will increase the OS further. In both low and high levels of initial OS conditions, aging will affect this process negatively by increasing OS, such as increasing mtDNA mutation accumulation and decline in mitochondrial functions. Under increasing OS conditions with aging, individuals with DS will be prone to develop more morbid conditions and prone to death depending on their initial adaptive response signaling. In other words, negative factors will lead to earlier clinical manifestations of age-related conditions, while positive adaptations (e.g., conditioned hormetic signaling) may support normal cellular and systemic functions for longer periods of time.

In conclusion, DS is the result of a whole genome imbalance caused by triplication of HSA21 genes. The severity and spectrum of the syndrome vary greatly. Besides oxidative damage, mtDNA mutations and mitochondrial dysfunction emerge as important modulators of DS phenotypes. This variability is further influenced by an adaptive cellular response to stress. A comprehensive and detailed analysis of signature pathways unique to hormesis will be required to fully assess the role of the adaptive response in DS (Figure 3). Key elements of hormesis may be valuable predictors of disease onset and treatment outcomes in DS individuals.
